# Obstructive Sleep Apnea in Patients with Significant Coronary Artery Disease: An Underdiagnosed Condition

**DOI:** 10.3390/jcm15082877

**Published:** 2026-04-10

**Authors:** Monika Kowalik-Pandyra, Klaudia Piwowar, Michał Tworek, Larysa Bielecka, Małgorzata Mazur, Anna Kabłak-Ziembicka, Jakub Podolec

**Affiliations:** 1 Department of Cardiac and Vascular Diseases, St. John Paul II Hospital, Prądnicka 80, 31-202 Kraków, Poland; monika.kowalik@doctoral.uj.edu.pl (M.K.-P.); michal.tworek@doctoral.uj.edu.pl (M.T.); larysabielecka@gmail.com (L.B.); m.mazur@szpitaljp2.krakow.pl (M.M.); kablakziembicka@op.pl (A.K.-Z.); 2 Doctoral School of Medical and Health Sciences, Jagiellonian University Medical College, Łazarza 16, 31-530 Kraków, Poland; 3 Department of Interventional Cardiology, Institute of Cardiology, St. John Paul II Hospital, Prądnicka 80, 31-202 Kraków, Poland; k.piwowar@szpitaljp2.krakow.pl; 4 Department of Cardiac and Vascular Diseases, Institute of Cardiology, Jagiellonian University Medical College, Św. Anny 12, 31-007 Kraków, Poland

**Keywords:** Apnea–Hypopnea Index, coronary artery disease, obstructive sleep apnea, oxygen desaturation index, SYNTAX score, risk factors

## Abstract

**Background**: Obstructive sleep apnoea (OSA) is a highly prevalent yet underdiagnosed disorder in patients with cardiovascular disease. Growing evidence suggests a pathophysiological link between OSA and coronary artery disease (CAD); however, the relationship between OSA severity and anatomical complexity of coronary lesions remains incompletely understood. **Aim**: The aim of this study is to assess the prevalence of OSA in patients undergoing coronary angiography and to evaluate the association between sleep-disordered breathing parameters and the severity of CAD expressed by the SYNTAX score. **Methods**: This prospective study enrolled 103 consecutive patients referred for invasive coronary angiography. All participants underwent overnight type III cardiorespiratory polygraphy. OSA severity was classified according to the Apnea–Hypopnea Index (AHI). The anatomical complexity of CAD was assessed using the SYNTAX score. Linear regression analyses were performed to determine associations between polysomnographic parameters and SYNTAX score. **Results**: Significant CAD was diagnosed in 74.8% of patients. OSA was highly prevalent, with severe OSA observed in 36.4% of patients with significant CAD compared to 3.8% in those without significant stenoses (*p* = 0.003). Patients with significant CAD had higher AHI (18.8 vs. 13.5 events/h; *p* = 0.003), higher oxygen desaturation index (ODI) (19.3 vs. 12.9 events/h; *p* = 0.003), and greater mean oxygen desaturation (4.1% vs. 3.8%; *p* = 0.008). In multivariable regression analysis, AHI (B = 0.329; 95% CI [0.083, 0.576]; *p* = 0.009) and nicotinism (B = 8.693; 95% CI [2.573, 14.814]; *p* = 0.006) independently predicted higher SYNTAX scores. Interestingly, each 1% increase in snoring percentage was associated with a 0.203-point reduction in SYNTAX score (95% CI [−0.339, −0.068]; *p* = 0.004). **Conclusions**: OSA is highly prevalent in patients undergoing coronary angiography and is independently associated with greater anatomical complexity of CAD. Sleep-disordered breathing, particularly AHI and nocturnal hypoxemia, may represent important non-traditional risk markers of advanced coronary atherosclerosis. Systematic screening for OSA should be considered in patients with suspected or confirmed CAD.

## 1. Introduction

Coronary artery disease (CAD) remains one of the leading causes of morbidity and mortality worldwide [1]. Epidemiological data indicate that the prevalence of CAD increases markedly with age, reaching approximately 20% among adults aged 75 years and older [2]. Its development results from the interplay between genetic susceptibility and modifiable cardiovascular risk factors [3].

According to the European Society of Cardiology (ESC), the major determinants for CAD include arterial hypertension, diabetes mellitus, dyslipidemia, cigarette smoking, obesity, and a family history of premature cardiovascular disease [1]. These conditions are defined by established diagnostic criteria, such as elevated blood pressure, abnormal glucose metabolism, lipid abnormalities, or recent tobacco use [4].

Clinical suspicion of CAD typically arises in patients reporting exertional chest pain or related symptoms, with severity assessed using the Canadian Cardiovascular Society (CCS) classification [1]. Non-invasive evaluation includes exercise treadmill testing, the identification of regional wall-motion abnormalities on echocardiography, or elevated coronary artery calcium (CAC) scores, all of which increase the likelihood of obstructive CAD [1,5,6]. Definitive diagnosis relies on invasive coronary angiography, which quantifies the severity of stenosis and guides decisions regarding revascularization. Significant CAD is defined as ≥50% stenosis in the left main coronary artery or ≥70% stenosis in major epicardial branches [1]. Revascularization strategies include percutaneous coronary intervention (PCI) or coronary artery bypass grafting (CABG), with the choice depending on anatomic complexity-expressed by the SYNTAX score-and clinical factors such as diabetes or multivessel CAD [1,7,8,9].

Besides classic risk factors of CAD, there are many clinical and biochemical risk factors that initiate atherosclerosis progression leading to major adverse cardiac and cerebrovascular events (MACCE) [10]. Among others, obstructive sleep apnea (OSA) is an underdiagnosed disorder which may share pathophysiological pathways with coronary atherosclerosis and potentially contribute to MACCE [11].

OSA is characterized by recurrent upper-airway obstruction during sleep, leading to intermittent hypoxia, hypercapnia, and sleep fragmentation [12].

OSA significantly reduces quality of life (QoL) in terms of physical, social, and emotional functioning, especially in moderate and severe forms [13]. Furthermore, OSA is independently associated with increased cardiovascular risk and higher cardiovascular mortality [14,15].

Globally, nearly one billion adults may be affected, including a substantial proportion with moderate-to-severe disease [16]. In Poland, epidemiological data suggest that up to two million adults may meet diagnostic criteria for OSA, although most cases remain unrecognized [17].

It is believed that OSA may worsen outcomes in cardiovascular disease [18]. It can occur in 30–50% of patients with resistant hypertension, up to 30% with CAD, and nearly half of those with chronic heart failure [19,20]. The diagnosis of OSA may impose technical difficulties due to limited availability of diagnostic equipment. However, overnight polysomnography (PSG) is considered the gold standard to confirm OSA with recordings of the number and duration of apnea episodes, snoring, and oxygen saturation during sleep [21]. While overnight polysomnography (PSG) is considered the gold standard, type III cardiorespiratory polygraphy is a validated alternative for OSA screening in clinical settings.

The present study aimed to evaluate the prevalence of OSA in patients diagnosed for CAD, and whether CAD severity expressed as SYNTAX score correlates with OSA findings.

## 2. Materials and Methods

### 2.1. Subjects

This study enrolled 103 consecutive patients referred for coronary angiography with suspected CAD in whom overnight polygraphy was performed during the hospital stay.

Inclusion criteria were age ≥ 18 years, clinical indication for coronary angiography, and written informed consent. The study exclusion criteria included history of malignancy or autoimmune disease, severe or uncontrolled pulmonary disease (including pulmonary hypertension, chronic obstructive pulmonary disease, asthma), a previous diagnosis of sleep apnea, pregnancy or breastfeeding, evidence of active infection and lack of clinical indications for invasive coronary angiography.

Patients were divided into two groups according to the presence of significant CAD [1]. Demographic characteristics, anthropometric data, comorbidities, and laboratory results were collected from medical records. A history of myocardial infarction was not considered an exclusion criterion. Fasting blood samples were analyzed for lipid profile, glucose, hsCRP, and NT-proBNP. Echocardiography was performed according to current ESC/ASE recommendations to assess LVEF and diastolic parameters. Echocardiographic data were available for all included patients.

All subjects gave their informed consent prior to enrolment in accordance with the requirements of the institutional local Ethics Committee (No. 1072.6120.203.2022). The study was performed consistent with the requirements of the Declaration of Helsinki.

### 2.2. Cardiorespiratory Polygraphy

Monitoring was performed using a ResMed type III cardiorespiratory polygraph (type III according to the American Academy of Sleep Medicine, AASM, ResMed, San Diego, CA, USA). Parameters included airflow, thoracoabdominal movements, oxygen saturation, and body position. Overnight cardiorespiratory polygraphy was performed after patient inclusion into the study and after obtaining written informed consent, during hospitalization. The Apnea–Hypopnea Index (AHI) and Oxygen Desaturation Index (ODI) were calculated. Apnea–Hypopnea Index (AHI) was calculated based on total recording time (REI according to AASM guidelines), as sleep time is not available in type III studies. The Apnea–Hypopnea Index (AHI), defined as the number of apnea and hypopnea events per hour of recording (REI), was used to classify OSA severity. OSA severity was categorized as mild (5–14.9 events/h), moderate (15–29.9 events/h), or severe (≥30 events/h) [12]. Sleepiness was assessed using the Epworth Sleepiness Scale (ESS), and risk of OSA with the STOP-BANG questionnaire. Subjective daytime sleepiness was evaluated using the Epworth Sleepiness Scale (ESS), a self-administered questionnaire where patients rate their likelihood of falling asleep in eight common daily situations on a scale from 0 to 3. The total score ranges from 0 to 24, with a score exceeding 10 points defined as excessive daytime sleepiness [22]. To determine the clinical risk of obstructive sleep apnea, the STOP-BANG questionnaire was utilized. This screening tool incorporates four subjective symptoms (snoring, tiredness, observed apnea, and high blood pressure) and four objective demographics (body mass index > 35 kg/m^2^, age > 50 years, neck circumference > 40 cm, and male sex), with a score of 3 or more points signifying an increased risk of OSA [23]. Mean oxygen desaturation was defined as the average depth of all desaturation events associated with respiratory disturbances.

### 2.3. Coronary Angiography and SYNTAX Score

All patients underwent diagnostic coronary angiography using standard techniques. The diagnosis of coronary artery disease and the assessment of myocardial ischemia were based on clinical presentation, noninvasive assessment, and invasive coronary angiography performed in all patients in accordance with current ESC clinical guidelines. Coronary lesions were assessed by independent cardiologists blinded to the sleep study results. The anatomical complexity of CAD was evaluated using the SYNTAX score. The original SYNTAX score was used as it reflects the anatomical complexity of coronary artery disease. The use of SYNTAX II, which includes clinical variables, could introduce overlap with factors already included in regression models. Based on the original validation of the score, patients were classified into a group with low anatomical complexity (SYNTAX score ≤ 22) and a group with moderate or high anatomical complexity (SYNTAX score > 22) [1]. The cutoff value of 22 was based on the results of the SYNTAX study, which demonstrated significant prognostic and treatment differences between these groups. Due to the analytical design and the size of the study population, patients with moderate and high anatomical complexity were analyzed as a single group, an approach commonly adopted in studies using the SYNTAX score. Noninvasive assessment was performed prior to hospital admission in the outpatient setting and included exercise stress testing, echocardiographic evaluation of wall-motion abnormalities, and/or coronary artery calcium scoring, depending on clinical indication. These data were used as part of the clinical decision-making process for referral to coronary angiography and were not systematically collected for the purpose of this study.

### 2.4. Statistical Analyses

The data were collected in a dedicated database and analyzed statistically. Data distribution was assessed using the Kolmogorov–Smirnov test. Depending on distribution, group comparisons were performed using Student’s *t*-test (with Welch’s correction), the Mann–Whitney U test, or the chi-square test. Univariable and multivariable linear regression analyses were performed to evaluate the association between the SYNTAX score and clinically relevant variables. Additional variables, including the STOP-BANG score and hsCRP concentration, were analyzed in separate models. A *p*-value < 0.05 was considered statistically significant. Variables significant in univariable linear regression analysis (*p* < 0.05) were included in the multivariable linear regression model. All analyses were performed using IBM SPSS Statistics version 29 (IBM Corp., Armonk, NY, USA). Model fit was assessed using Nagelkerke R^2^ where applicable.

## 3. Results

### 3.1. Study Population and Clinical Characteristics

**Figure 1:**
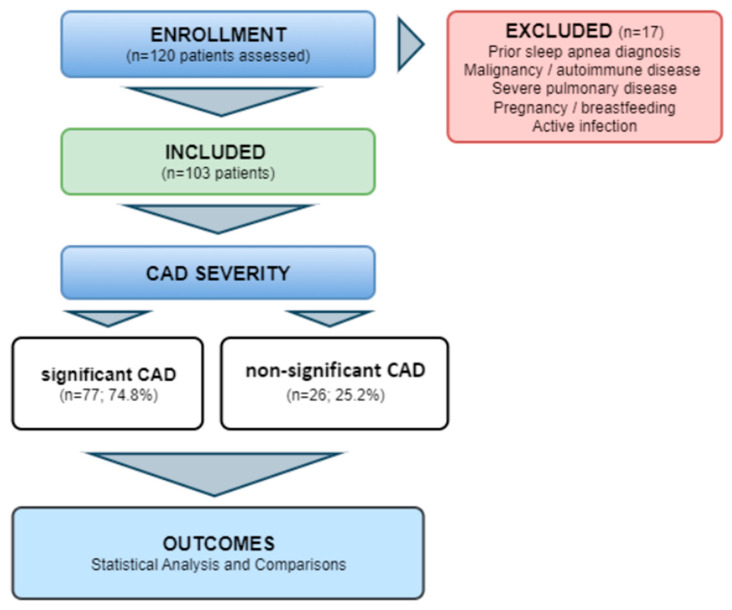
Methodological flowchart of the study.

Out of initially analyzed 120 patients, a total of 103 patients were included in the study, of whom 77 (74.8%) had significant CAD and 26 (25.2%) had non-significant CAD. Study flowchart is presented in Figure 1.

The mean age did not differ significantly between the groups (68 [Q1 63; Q3 72] vs. 68 [Q1 61; Q3 71] years, *p* = 0.551). Male gender was predominant among patients with significant CAD (84.4% vs. 61.5%, *p* = 0.014). No significant differences were observed in body weight, height, or BMI between the groups. Hypertension and dyslipidemia were the most prevalent chronic conditions in both groups, with no statistically significant differences (*p* > 0.05). The prevalence of type 2 diabetes, chronic kidney disease, smoking, and heart failure did not differ significantly; however, left ventricular ejection fraction was significantly lower in the CAD group (*p* = 0.002).

Median hsCRP levels were significantly higher in the CAD group compared with the non-significant CAD group (1.44 [Q1 0.79; Q3 2.20] vs. 0.77 [Q1 0.51; Q3 1.69] mg/L; *p* = 0.019). Whereas total cholesterol, LDL cholesterol and HDL cholesterol levels were significantly lower in CAD group. NT-proBNP levels showed no statistically significant difference between groups (*p* = 0.298). The detailed study groups characteristics are presented in Table 1.

### 3.2. Baseline Characteristics of Sleep-Disordered Breathing Parameters: OSA Severity

Of a total of 103 patients, cardiorespiratory polygraphy revealed significant disparities in the severity of sleep-disordered breathing between the study groups. The distribution of OSA severity showed that only 3.9% of patients with significant CAD were free of the disorder, while 36.4% suffered from severe OSA. In contrast, 19.2% of patients with non-significant CAD had no OSA, and only 3.8% were classified as severe (*p* = 0.003). Mild and moderate OSA were found in 33.8% and 26.0% of the significant CAD group, respectively, compared to 42.3% and 34.6% in the non-significant group. These findings were reflected in the ODI, which was significantly higher in patients with significant CAD (19.30 vs. 12.85; *p* = 0.003), as well as in the mean oxygen desaturation levels (4.10% vs. 3.80%; *p* = 0.008). Clinical screening via the STOP-BANG scale showed higher scores in the significant CAD group (5.00 vs. 4.00; *p* = 0.005), while subjective daytime sleepiness assessed by the Epworth Sleepiness Scale was more intense in these patients, with 10.4% reporting severe symptoms compared to 0.0% in the non-significant group (*p* = 0.007).

The detailed characteristics of sleep-disordered breathing parameters are included in Table 2.

### 3.3. Sleep-Disordered Breathing Parameters Depending on the CAD Severity

**Figure 2:**
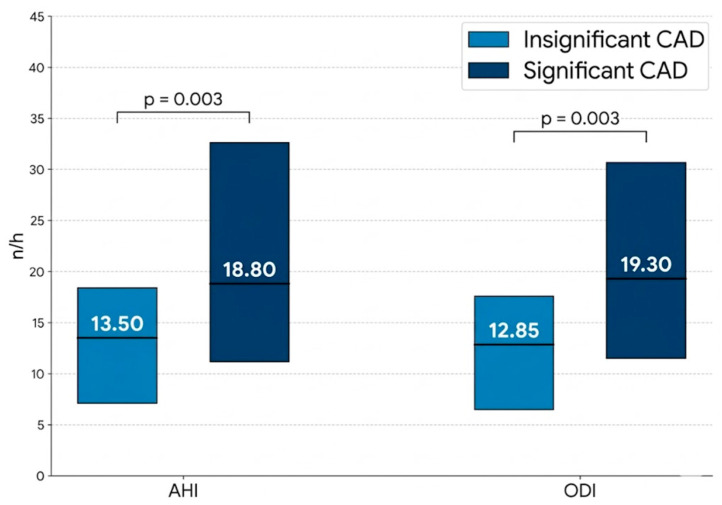
Comparison of Sleep-Disordered Breathing Parameters according to the CAD severity.

The severity of sleep-disordered breathing was significantly more pronounced in patients with significant CAD compared to the non-significant CAD group. Specifically, patients with CAD exhibited a significantly higher AHI (18.80 (11.20–32.60) vs. 13.50 (7.10–18.40) [n/h], median (IQR); *p* = 0.003) and a higher ODI 19.30 (11.50–30.70) vs. 12.85 (6.50–17.60) [n/h], median (IQR); *p* = 0.003 (Figure 2).

Furthermore, the clinical severity of OSA was significantly higher in patients with CAD. Severe OSA was significantly more frequent in patients with CAD compared to the non-CAD group (36.4% vs. 3.8%; *p* = 0.003). This was further supported by respiratory data: the mean oxygen desaturation was significantly greater in the CAD group (4.1 (3.7–4.9) vs. 3.8 (3.4–4.2) [%], median (IQR); *p* = 0.008). Subjective and objective screening also reflected this trend. Patients with CAD scored significantly higher on the STOP-BANG scale (5.00 (4.00–6.00) vs. 4.00 (3.00–4.00) [pts], median (IQR); *p* = 0.005) and reported higher daytime sleepiness, as assessed by the Epworth Sleepiness Scale (*p* = 0.007).

Analysis of oxygenation parameters revealed that the mean oxygen desaturation drop was more severe in patients with significant coronary lesions (4.10 (3.70–4.90) vs. 3.80 (3.40–4.20) [%], median (IQR); *p* = 0.008).

### 3.4. Association of Polysomnographic Parameters, Gender, and Nicotinism with SYNTAX Score in Univariate Linear Regression Analysis

In the univariate linear regression analysis of polysomnographic parameters, a statistically significant correlation was demonstrated between the AHI score, percentage of snoring, mean drop in saturation, and SYNTAX score (*p* < 0.05). Each 1-point increase in AHI increased the SYNTAX score by 0.360 (95% CI [0.148, 0.572]), and each 1-point increase in the average drop in saturation increased the score by 3.725 (95% CI [0.946, 6.503]). In turn, each 1% increase in snoring reduced the SYNTAX score by 0.147 (95% CI [−0.296, −0.002]). Additionally, a statistically significant relationship between gender and nicotinism and the SYNTAX score was demonstrated for the remaining parameters (*p* < 0.05). Male gender increased the SYNTAX score by 8.821 (CI 95% [0.792, 16.851]) and nicotinism by 7.824 (CI 95% [1.324, 14.325]). The residuals have an expected value of 0, homoscedasticity was verified based on an analysis of the scatter plot of residuals against predicted values (Table 3).

### 3.5. Independent Predictors of SYNTAX Score in Multiple Linear Regression Analysis

Statistically significant variables from the single linear regression model were introduced into the multiple linear regression model. The analyzed model is statistically significant (*p* < 0.001). The coefficient of determination R2 was calculated at 0.317, which indicates that the presented model explains 31.7% of the variability in SYNTAX scores in patients with significant coronary artery disease. The regression coefficient R was 0.563, F 6.599, df 5. No collinearity was observed in the model (VIF: 1.620, 1.116, 1.612, 1.098, 1.140, respectively). Residuals were normally distributed with a mean of approximately zero, homoscedasticity was verified based on the analysis of the scatter plot of residuals against predicted values, no autocorrelation was found (Durbin–Watson 1.702), each of the random components has a normal distribution.

A statistically significant correlation was demonstrated between the AHI score, snoring percentage, and nicotinism and the SYNTAX score (*p* < 0.05). Each 1-point increase in AHI resulted in a 0.329 increase in SYNTAX score (95% CI [0.083, 0.576]), and each 1% increase in snoring reduced SYNTAX score by 0.203 (95% CI [−0.339, −0.068]). In addition, a statistically significant relationship between nicotinism and the SYNTAX score was demonstrated. Nicotinism increased the SYNTAX score by 8.693 (95% CI [2.573, 14.814]) (Table 4).

### 3.6. Echocardiographic Characteristics and Functional Assessment

Echocardiographic analysis revealed significant differences in cardiac structure and function between the study groups. Patients with significant coronary artery disease (CAD) had significantly lower left ventricular ejection fraction (LVEF) compared to those without significant CAD (55.0% [46.0; 60.0] vs. 61.0% [55.0; 65.0], *p* = 0.002).

In addition, patients in the CAD group presented with larger left ventricular dimensions, both in systole (LVESD: 36.00 mm vs. 30.50 mm, *p* = 0.002) and diastole (LVEDD: 52.2 mm vs. 46.7 mm, *p* = 0.009), indicating the presence of adverse ventricular remodeling.

No significant differences were observed between the groups in right ventricular systolic pressure (RVSP) or left atrial volume index (LAVi). Detailed echocardiographic parameters are presented in Table 5.

## 4. Discussion

### 4.1. OSA Severity and CAD Severity

In this study, we found a significant association between obstructive sleep-disordered breathing and coronary atherosclerosis, both in terms of presence and anatomical extent, as measured by higher SYNTAX scores in patients with more severe sleep apnea. Our findings align with previous evidence supporting the role of OSA as a non-traditional cardiovascular risk factor in the setting of CAD [24]. We have observed that patients with significant CAD had higher AHI and ODI values and were more likely to have severe OSA. The results of our study were like those of Zhang et al., who demonstrated a positive correlation between SYNTAX scores and OSA severity [25]. Among the patients studied, groups with high burden of nocturnal hypoxia had significantly higher SYNTAX scores and an independently higher risk of severe CAD [25]. Ishiwata et al. showed that patients with moderate-severe OSA had significantly higher SYNTAX, which also confirms our results [26]. Multivariate analysis in our study revealed that AHI and smoking were an independent predictor of higher SYNTAX scores, even after adjustment for traditional cardiovascular risk factors such as age, sex, hypertension, diabetes, dyslipidemia.

An unexpected finding of our study was the inverse association between snoring percentage and SYNTAX score. Although seemingly paradoxical, snoring itself may not accurately reflect the severity of obstructive events or the burden of intermittent hypoxia. More advanced OSA, particularly in patients with complex CAD, may be characterized by prolonged complete airway collapse with less prominent acoustic vibration but greater oxygen desaturation [25,26]. Previous studies have demonstrated that hypoxic burden correlates more strongly with coronary atherosclerosis severity than snoring frequency alone [25,27]. As emphasized in the guidelines of the European Society of Cardiology, endothelial dysfunction and inflammation play a central role in atherosclerosis progression [1]. Therefore, AHI and desaturation indices appear to be more reliable markers of CAD complexity than snoring percentage.

STOP-BANG is a commonly used screening tool; however, some studies suggest that this scale does not always show a significant correlation with the severity of CAD assessed by SYNTAX score, likely due to the limitations of these screening methods in predicting CAD burden [28]. At the same time, the literature emphasizes that full polysomnography, including assessment of nocturnal hypoxic burden, provides a more accurate reflection of the relationship between OSA and the anatomical complexity of coronary atherosclerosis [27]. These findings should be interpreted with caution due to the observational nature of the study.

In addition, poor sleep quality is more frequently observed in patients presenting with higher SYNTAX scores, accounting for nearly 43% of patients [29].

This highlights a potential role of sleep-disordered breathing beyond established risk markers.

### 4.2. The Role of OSA in CAD Pathogenesis

The pathophysiology of OSA involves interactions between mechanical obstruction of the upper airway and systemic physiological responses. During sleep, reduced tone of the pharyngeal dilator muscles leads to airway collapse, causing episodes of apnea or hypopnea. These result in cyclic oxygen desaturation and reoxygenation, generating oxidative stress via reactive oxygen species (ROS) [30]. Recurrent intermittent hypoxia activates transcription factors including hypoxia-inducible factor-1α (HIF-1α) and nuclear factor-κB (NF-κB), promoting release of proinflammatory cytokines (IL-6, TNF-α, CRP) and expression of adhesion molecules (VCAM-1, ICAM-1) [31]. That pathological pathway leads to many cardiovascular disorders showing interplay between respiratory and cardio-cerebro-vascular pathological mechanism [32,33,34].

The cascade produces systemic inflammation, endothelial dysfunction, and increased arterial stiffness, which shares accelerating atherogenesis and plaque progression in coronary and peripheral arteries [35,36].

Repeated arousals and hypoxemia stimulate sympathetic nervous system activation, leading to transient surges in blood pressure, tachycardia, and increased myocardial oxygen demand [37,38,39].

Chronic sympathetic overactivity contributes to hypertension, insulin resistance, dyslipidemia, and adverse cardiac remodeling [40,41].

Intermittent hypoxia also impairs nitric oxide (NO) bioavailability, reducing vasodilation and further damaging vascular endothelium [42]. These mechanisms may collectively contribute to more severe CAD in patients with untreated OSA.

However, it remains debated whether the pathophysiological mechanisms triggered by OSA directly accelerate atherosclerosis, or whether OSA and CAD progress in parallel via shared pathways [14,43]. For example, low-grade systemic inflammation is common to both conditions, and concurrent activation of the NLRP3 inflammasome pathway may underlie elevated IL-1β and IL-6 levels in both OSA and CAD [44,45]. Similarly, matrix metalloproteinase-9 (MMP-9) activation may link OSA to plaque instability and rupture [46].

Endothelial activation and related prothrombotic pathways may further exacerbate vascular dysfunction, as demonstrated in other vascular conditions such as idiopathic pulmonary arterial hypertension [47]. If specific mechanisms of OSA contribute to the activation of more targeted pathways for atherosclerosis progression, particular attention should be given to increased endothelin secretion and oxidative stress, which can further exacerbate endothelial dysfunction and promote plaque development [48]. Evidence also suggests that OSA may contribute to plaque vulnerability, potentially increasing the risk of cardiovascular events two-fold. Mechanisms proposed include endothelial dysfunction through peroxynitrite accumulation in microvascular walls, chronic hypoxia-induced inflammation, and oxidative stress, all of which may precipitate CAD development and progression [49]. These observations are further supported by recent work highlighting the role of coronary microvascular dysfunction in patients with chronic coronary syndromes. Legutko et al. demonstrated that microvascular impairment contributes to discordance between fractional flow reserve (FFR) and resting full-cycle ratio (RFR), indicating that factors beyond epicardial stenosis can influence the functional and anatomical assessment of CAD [50]. In the context of OSA, recurrent intermittent hypoxia, endothelial dysfunction, and systemic inflammation may similarly affect the coronary microcirculation, potentially exacerbating anatomical complexity and plaque vulnerability. These findings suggest that the relationship between OSA severity and CAD may involve both macrovascular atherosclerosis and microvascular mechanisms, underlining the importance of comprehensive cardiovascular evaluation in patients with sleep-disordered breathing. In addition to microvascular dysfunction, anatomical coronary variants may further influence the relationship between OSA and CAD. Data from the National Polish Percutaneous Interventions Registry indicate that myocardial bridges are not uncommon and may modify coronary flow dynamics, potentially predisposing to ischemia under conditions of increased myocardial demand and hypoxia, such as in severe OSA [51]. While the exact causal relationships remain uncertain, these findings support the concept that OSA can exacerbate the atherosclerotic process, either through direct mechanistic effects or via overlapping inflammatory and oxidative pathways shared with CAD.

### 4.3. Clinical Correlations and Implications

In our present study, a higher degree of sleep apnea was more frequent in men. In line, Sasanabe et al. [52] showed a statistically significantly higher number of men, which may suggest the need to pay more attention to active search for OSA in this group of patients.

Several studies have considered the potential impact of low-grade chronic inflammation, which may result in increased coronary atherosclerosis and, as a consequence, in CAD and acute coronary syndromes [53].

A study by Shobatake et al. observed the negative consequences of intermittent hypoxemia, a characteristic manifestation of OSA [54]. It was shown that hypoxemia can affect cellular metabolism, which can lead to impaired glucose tolerance, insulin resistance, hypertension and cardiovascular dysfunction. The phenomenon obliges us to evaluate the average drop in saturation and counteract by including effective treatment for OSA. The mechanisms identified in the studies for the co-occurrence of IHS and OSA are intermittent hypoxia, oxidative stress, sympathetic activation and endothelial dysfunction [55].

The observed reduction in left ventricular ejection fraction (LVEF) and the presence of ventricular enlargement in patients with significant CAD likely reflect the cumulative effects of chronic myocardial ischemia and a higher prevalence of prior myocardial infarction in this group. These findings are consistent with contemporary evidence indicating that coronary artery disease is frequently associated with impaired myocardial function and adverse ventricular remodeling [56].

Importantly, these structural and functional alterations may contribute to the increased severity of obstructive sleep apnea (OSA) observed in our study. Patients with significant CAD presented with higher values of the Apnea–Hypopnea Index (AHI) and oxygen desaturation index (ODI), suggesting a clinically relevant interaction between cardiac dysfunction and sleep-disordered breathing.

The relationship between cardiac dysfunction and OSA appears to be bidirectional. On one hand, reduced cardiac output and altered ventricular mechanics may promote ventilatory instability and predispose to sleep-disordered breathing. On the other hand, recurrent intermittent hypoxia, sympathetic activation, and intrathoracic pressure swings associated with OSA may further impair myocardial function and contribute to adverse cardiac remodeling [12,20].

Although not all echocardiographic parameters (such as SV, CI, or PAWP) were available for the entire cohort, the observed differences in LVEF and ventricular dimensions indicate a greater functional burden in patients with significant CAD, which may partially explain the higher severity of OSA in this group.

Taken together, these findings support the concept of a complex interaction between coronary artery disease severity, myocardial dysfunction, and obstructive sleep apnea, in which both conditions may mutually reinforce each other and contribute to increased cardiovascular risk.

### 4.4. Study Limitation

This is a prospective single-center study in which we tried to ensure the highest quality of data. The use of type III cardiorespiratory polygraphy instead of full polysomnography may have led to an underestimation of AHI. The limited number of patients in our study may pose a potential problem in the statistical interpretation of the results. The inclusion of patients with a prior history of myocardial infarction may have influenced the assessed severity of coronary artery disease. Developing statistical models on such a small group of patients can be problematic, especially when the number of variables is important. However, the level of statistical significance was sufficient to support the conclusions presented. The assessment of obstructive sleep apnea using portable monitoring has inherent limitations; however, the development of results by experienced medical personnel allows us to ensure the highest quality results. Noninvasive test results were not systematically collected, which may have limited additional analyses. Pharmacological treatment (e.g., statins, ACE inhibitors, beta-blockers) was not included in regression models and may have influenced biochemical and echocardiographic parameters. The relatively small sample size limited the number of variables that could be included in multivariable models without increasing the risk of overfitting. Pharmacological treatment was not included in the analysis, which may have influenced cardiovascular parameters. Additionally, echocardiographic functional parameters were not incorporated into regression models and should be addressed in future studies. Given the modest sample size, the inclusion of numerous covariates in multivariable models may increase the risk of overfitting and reduce the robustness of regression coefficients. Consequently, multivariate results should be interpreted with appropriate caution. Despite the relatively small sample size, the study population was well-characterized, and the results provide strong clinical signals regarding the impact of OSA on coronary anatomy.

## 5. Conclusions

In conclusion, OSA is highly prevalent among patients undergoing coronary angiography and remains substantially underdiagnosed in this high-risk population. The severity of sleep-disordered breathing, particularly as reflected by AHI and indices of nocturnal hypoxemia, is independently associated with greater anatomical complexity of coronary artery disease assessed by the SYNTAX score.

These findings reinforce the concept that OSA represents an important non-traditional cardiovascular risk factor and may contribute to the progression of coronary atherosclerosis beyond established risk determinants such as age, smoking, hypertension, or diabetes. Although the inverse relationship observed between snoring percentage and SYNTAX score requires further validation, our results underscore the importance of comprehensive sleep evaluation rather than reliance on isolated symptoms.

Routine screening for OSA in patients with suspected or confirmed CAD may improve risk stratification and potentially open new avenues for integrated cardiopulmonary management strategies. Further large-scale prospective studies are needed to determine whether effective treatment of OSA can modify coronary plaque progression and improve cardiovascular outcomes.

## Figures and Tables

**Table 1:** Baseline demographic, clinical, and biochemical characteristics of the study population.

Parameter	All Study Participants (n = 103)	CAD Group (n = 77)	Non-CAD Group (n = 26)	*p*-Value
Age [years], median (IQR)	68 (62–72)	68 (63–72)	68 (61–71)	0.551
Male gender, n (%)	81 (78.6%)	65 (84.4%)	16 (61.5%)	0.014
Weight [kg], mean ± SD	86.0 ± 14.3	85.1 ± 11.9	88.6 ± 19.4	0.391
BMI [kg/m^2^], median (IQR)	28.5 (26.5–31.8)	28.34 (26.5–30.9)	29.74 (26.5–35.0)	0.127
Hypertension, n (%)	92 (89.3%)	71 (92.2%)	21 (80.8%)	0.103
Diabetes mellitus type 2, n (%)	31 (30.1%)	22 (28.6%)	9 (34.6%)	0.561
Previous MI, n (%)	21 (20.4%)	21 (27.3%)	0 (0.0%)	<0.001
Smoking, n (%)	25 (24.3%)	21 (27.3%)	4 (15.4%)	0.222
Dyslipidemia, n (%)	95 (92.2%)	71 (92.2%)	24 (92.3%)	0.987
Atrial Fibrillation, n (%)	17 (16.5%)	10 (13.0%)	7 (26.9%)	0.127
**Baseline biochemical data**				
Glucose [mmol/L], median (IQR)	5.60 (5.20–6.10)	5.70 (5.20–6.20)	5.40 (5.20–6.00)	0.815
Total cholesterol [mmol/L], median (IQR)	3.42 (2.86–4.00)	3.20 (2.76–3.67)	4.10 (3.58–4.68)	0.010
LDL cholesterol [mmol/L], median (IQR)	1.87 (1.35–2.41)	1.68 (1.27–2.04)	2.37 (1.79–3.02)	0.012
HDL cholesterol [mmol/L], median (IQR)	1.18 (0.98–1.40)	1.14 (0.97–1.37)	1.40 (1.14–1.48)	0.014
hsCRP [mg/L], median (IQR)	1.25 (0.71–2.14)	1.44 (0.79–2.20)	0.77 (0.51–1.69)	0.019
NT-proBNP [pg/mL], median (IQR)	148.0 (84.0–420.0)	168.0 (86.0–480.0)	123.5 (81.5–210.5)	0.298

Abbreviations: IQR—interquartile range, n—number, SD—standard deviation, BMI—body mass index, LDL—low-density lipoprotein, HDL—high-density lipoprotein, hsCRP—high-sensitivity C-reactive protein, NT-proBNP—N-terminal pro b-type natriuretic peptide.

**Table 2:** Respiratory and sleep parameters depending on the CAD severity.

Parameter	Patients with Non-Significant CAD [n = 26]	Patients with Significant CAD [n = 77]	*p*-Value
AHI [n/h], median (IQR)	13.50 (7.10–18.40)	18.80 (11.20–32.60)	0.003
OSA Severity [n (%)]			0.003
-None (<5 events/h)	5 (19.2%)	3 (3.9%)	
-Mild (5–14.9 events/h)	11 (42.3%)	26 (33.8%)	
-Moderate (15–29.9 events/h)	9 (34.6%)	20 (26.0%)	
-Severe (≥30 events/h)	1 (3.8%)	28 (36.4%)	
ODI [n/h], median (IQR)	12.85 (6.50–17.60)	19.30 (11.50–30.70)	0.003
Snoring percentage [%], median (IQR)	17.85 (9.80–27.80)	17.50 (8.50–32.00)	0.730
Mean oxygen desaturation [%], median (IQR)	3.80 (3.40–4.20)	4.10 (3.70–4.90)	0.008
Mean apnea duration [s], median (IQR)	16.05 (13.90–18.40)	17.90 (14.80–21.40)	0.093
Mean hypopnea duration [s], median (IQR)	21.70 (19.10–23.20)	23.20 (20.80–26.20)	0.054
STOP-BANG Scale [pts], median (IQR)	4.00 (3.00–4.00)	5.00 (4.00–6.00)	0.005
Epworth Sleepiness Scale [n (%)]			0.007
-Normal (0–10 pts)	15 (57.7%)	27 (35.1%)	
-Mild (11–14 pts)	10 (38.5%)	19 (24.7%)	
-Moderate (15–18 pts)	1 (3.8%)	23 (29.9%)	
-Severe (>18 pts)	0 (0.0%)	8 (10.4%)	

Abbreviations: IQR—interquartile range, n—number, AHI—Apnea–Hypopnea Index, ODI—Oxygen Desaturation Index, OSA—Obstructive Sleep Apnea, OSA severity defined according to AHI: mild 5–14.9, moderate 15–29.9, severe ≥ 30 [events/h].

**Table 3:** Univariate linear regression analysis of factors associated with the SYNTAX score.

	SYNTAX Score [pts]			
	B [95% CI]	Standard Error	Beta Standardized	*p*-Value
AHI [n/h]	0.360 [0.148, 0.572]	0.106	0.364	0.001
STOP-BANG [pts]	1.617 [−0.577, 3.811]	1.101	0.167	0.167
Epworth Sleepiness Scale [pts]	1.705 [−1.213, 4.624]	1.465	0.133	0.248
Snoring percentage [%]	−0.147 [−0.296, −0.002]	0.074	−0.227	0.047
Mean oxygen desaturation [%]	3.725 [0.946, 6.503]	1.395	0.295	0.009
Mean apnea duration [s]	0.455 [−0.044, 0.954]	0.251	0.205	0.073
Mean hypopnea duration [s]	0.123 [−0.581, 0.827]	0.353	0.040	0.729
Male gender	8.821 [0.792, 16.851]	4.031	0.245	0.032
Age [years]	0.072 [−0.288, 0.432]	0.181	0.046	0.691
Hypertension	7.036 [−4.053, 18.125]	5.566	0.144	0.210
Diabetes mellitus type 2	4.895 [−1.658, 11.449]	3.290	0.169	0.141
Smoking	7.824 [1.324, 14.325]	3.263	0.267	0.019
Dyslipidemia	−7.876 [−18.935, 3.183]	5.551	−0.162	0.160
Significant CAD	−2.602 [−10.862, 5.658]	4.146	−0.72	0.532
hsCRP [mg/L]	0.696 [−0.426, 1.818]	0.562	0.146	0.220

Abbreviations: n—number, CI—Confidence Interval, AHI—Apnea–Hypopnea Index, CAD—coronary artery disease, hsCRP—high-sensitivity C-reactive protein.

**Table 4:** Multivariate linear regression analysis of independent predictors of the SYNTAX score.

	SYNTAX Score [pts]			
	B [95% CI]	Standard Error (SE)	Beta (Standardized)	*p*-Value
AHI [n/h]	0.329 [0.083, 0.576]	0.123	0.333	0.009
Snoring percentage [%]	−0.203 [−0.339, −0.068]	0.068	−0.310	0.004
Mean oxygen desaturation [%]	0.793 [−2.344, 3.931]	1.574	0.063	0.616
Gender	4.097 [−3.281, 11.475]	3.700	0.114	0.272
Smoking	8.693 [2.573, 14.814]	3.069	0.296	0.006

Abbreviations: n—number, CI—Confidence Interval, AHI—Apnea–Hypopnea Index.

**Table 5:** Comparative characteristics of echocardiographic parameters in patients with insignificant coronary artery disease and patients with significant coronary artery disease.

Parameter	Patients with Non-Significant CAD [n = 26]	Patients with Significant CAD [n = 77]	*p*-Value
LVEDD [mm], mean ± SD	46.7 ± 3.5	52.2 ± 7.1	0.009
LVESD [mm], median (IQR)	30.50 (29.00–33.00)	36.00 (32.00–41.50)	0.002
LVEF [%], median (IQR)	61.00 (55.00–65.00)	55.00 (46.00–60.00)	0.002
RVOT prox [mm], mean ± SD	31.9 ± 4.7	32.7 ± 4.4	0.247
RVID1 [mm], mean ± SD	37.2 ± 5.7	38.3 ± 4.1	0.415
LA [mm], median (IQR)	40.50 (36.00–45.00)	42.00 (38.50–46.00)	0.126
LA area [cm^2^], median (IQR)	21.50 (18.00–24.00)	22.00 (21.00–24.00)	0.053
LAVi [mL/m^2^], median (IQR)	32.00 (25.00–36.00)	35.00 (30.00–38.00)	0.872
RA area [cm^2^], median (IQR)	17.75 (16.00–22.00)	18.00 (17.00–20.00)	0.168
RVSP [mmHg], mean ± SD	23.0 ± 7.0	17.8 ± 10.6	0.205

Abbreviations: LVEDD—left ventricular end-diastolic diameter; LVESD—left ventricular end-systolic diameter; LVEF—left ventricular ejection fraction; RVOT—right ventricular outflow tract, RVIDI1—right ventricular internal dimension; LA—left atrium; LAVi—left atrial volume index; RA—right atrium; RVSP—right ventricular systolic pressure; IQR—interquartile range; SD—standard deviation.

## Data Availability

The data is available on the request in justified circumstances.

## Abbreviations

The following abbreviations are used in this manuscript:

IQR	interquartile range
n	number
SD	standard deviation
BMI	body mass index
MI	myocardial infarction
ACS	acute coronary syndrome
LDL	low-density lipoprotein
HDL	high-density lipoprotein
hsCRP	high-sensitivity C-reactive protein
NT-proBNP	N-terminal pro b-type natriuretic peptide
AHI	Apnea–Hypopnea Index
ODI	Oxygen Desaturation Index
OSA	Obstructive Sleep Apnea
Q1	first quartile
Q3	third quartile
HFpEF	heart failure with preserved ejection fraction
HFrEF	heart failure with reduced ejection fraction
ICD	implantable cardioverter-defibrillator
CRT	cardiac resynchronization therapy
PCI	percutaneous coronary intervention
CABG	coronary artery bypass grafting
